# Sustainable upcycling of ABS plastic waste into nanostructured carbon for lithium-ion battery applications

**DOI:** 10.1039/d6ra04671a

**Published:** 2026-09-24

**Authors:** Aisha Zhanaikhan, Kuanysh Kurtibay, Aliya Mukanova, Sung-Soo Kim, Arailym Nurpeissova

**Affiliations:** a Institute of Batteries 53, Kabanbay Batyr Ave. Astana Kazakhstan; b Institute of New Materials and Energy Technologies, Nazarbayev University 53, Kabanbay Batyr Ave. Astana Kazakhstan arailym.nurpeissova@nu.edu.kz; c Chungnam National University 99 Daehak-ro Daejeon Republic of Korea

## Abstract

Acrylonitrile butadiene styrene (ABS), a high-performance thermoplastic widely used in electronics and 3D printing filaments, contributes significantly to the plastic waste problem due to its non-biodegradability. At the same time, the global push toward portable electronics and large-scale renewable energy storage systems has generated unprecedented demand for high-performance lithium-ion batteries (LIBs). We propose a way to convert ABS plastic waste directly from 3D printing scraps and failed prints into nanostructured carbon anodes for LIBs, valorizing a waste stream that is otherwise landfilled or downcycled. We systematically explored and compared the efficiency of our synthetic route, an acid-assisted route involving H_2_SO_4_ cross-linking prior to pyrolysis, with that of the conventional method. The addition of sulfonic groups (–SO_3_H) to the polymer structure stabilized the structure during heat treatment and promoted partial graphitization and the creation of a disordered porous structure. As a result, the carbon yield *via* cross-linking reached 30.13%, which is 6 times higher than that yielded by bare ABS. Structural characterization confirmed an expanded interlayer spacing of 3.7 Å, the presence of nanoscale graphitic domains, and a defect-rich microstructure, which are characteristics favorable for Li^+^ intercalation. In terms of electrochemical performance, the anode demonstrated a reversible capacity of more than 300 mAh g^−1^ in the first cycle, which was retained even after 100 cycles with a coulombic efficiency of 98%. It maintained excellent performance even at −15 °C. This work demonstrates that ABS plastic waste—one of the fastest-growing plastics in the 3D printing industry—can be directly converted into high-performance anode materials for lithium-ion batteries, offering a practical route for diverting this waste stream toward a valuable end use. A full assessment of the process's environmental footprint (chemical and energy inputs and wastewater generation) is needed before establishing its broader sustainability relative to the processes using conventional carbon sources.

## Abstract 2

ABS plastic waste is upcycled into nanostructured carbon anode through H_2_SO_4_-assisted crosslinking, increasing carbon yield by nearly sixfold. The resulting anode delivers ∼350 mAh g^−1^ after 100 cycles and exhibits stable low-temperature performance.

## Graphical abstract

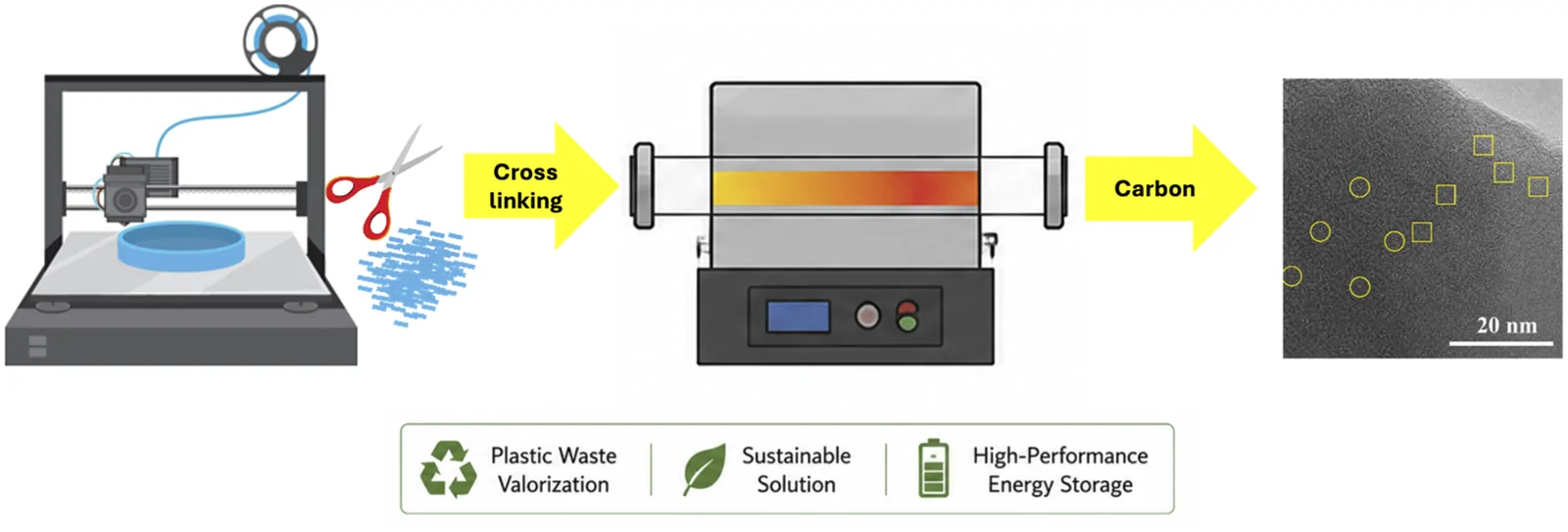

## Introduction

The world's population surge has reached its highest level, which has triggered rapid energy demand in terms of power and fuel. The current infrastructure is not efficient enough to meet the growing requirements. To tackle this situation, stakeholders are incorporating the use of all possible resources, such as solar photovoltaics and wind power, which are highly renewable and environmentally friendly.^1–6^ All these renewable resources are alternatives to fossil fuels, but they still lack the efficiency to reach the desirable goals.^7,8^ For instance, solar energy is available throughout daylight hours even when it is not needed, necessitating the storage of excess energy for use during peak demand hours.^9,10^ For this, we need advanced energy storage systems to efficiently provide the required amount of energy when it is needed.^11,12^ In this regard, several types of technologies are being used, among which lithium-ion batteries have the highest market share.^13,14^ Over 80% of the battery market is dominated by LIBs, and their major applications are in stationary storage systems and electric vehicles, with the rise of fuel prices around the world.^15,16^ Electric vehicles are considered one of the most promising solutions for reducing dependence on fossil fuels and meeting the growing demand for sustainable transportation.^17^ As we know, the cathode and anode materials of LIBs account for 40% and 18% of their cost, respectively, making these components important to be optimised.^18,19^ Most research is conducted on cathode materials, because graphite has been considered a universal anode material.^20,21^ Graphite is a crystalline, soft form of carbon, which is available in the Earth's crust, with an estimated resource of 300 million metric tonnes.^22,23^ However, graphite being a non-renewable source, scientists are looking for alternative carbons that can be generated from readily available sources.^24,25^ The world generates 2 billion tonnes of waste annually, of which 400 million tonnes are plastic.^26,27^ With rapid advancement in technologies, plastic waste has risen exponentially. Plastics themselves are non-renewable petroleum-generated products, and they are not degradable for centuries, which makes them problematic for global well-being.^28,29^ Globally, the 3R principle is utilised: reduce, reuse and recycle.^30^ Several alternatives to plastic have been introduced, but it still poses enormous problems to the world.^31^ There are several types of plastics, including PET, PVC, and ABS.^32^ Among the several million tonnes of plastic waste, 8 million tonnes are ABS waste, which comes mostly from the automotive industry, household appliances, and 3D printing.^33^ ABS plastic is a tough, rigid, and amorphous thermoplastic that deforms on heating and can be converted into a carbon material, which can be further used as a raw material for new components, especially electronics.^34^ To efficiently use ABS plastic waste, several studies have been published, but only a few have suggested to incorporate this carbonaceous material as an anode material in LIBs.^35^ Because the yield percentage is significantly low when it is carbonised, several techniques are utilised to tackle this problem.^36^

Research on the creation of carbon compounds from waste for use in LIB electrodes has advanced significantly in recent years.^37^ Wood, fruit peels, and agricultural waste are examples of biomass that have been thoroughly researched because they are inexpensive and renewable.^38^ Due to its high intrinsic carbon content and almost limitless availability from industrial and post-consumer streams, plastic waste, particularly those of polyethylene terephthalate (PET), polypropylene (PP), and polystyrene (PS), is also gaining attention as a carbon precursor.^39^ To break down volatile components and create a stable carbon matrix, most conversion techniques rely on high-temperature carbonization, which is usually done in an inert atmosphere at temperatures between 600 and 1000 °C.^40^ Chemical pre-treatments are frequently employed to modify the pore architecture, add functional groups and heteroatoms, and increase the carbon output.^41,42^ Because it can alter the polymer structure, encourage the development of char, and provide surface functional groups that directly affect the electrochemical performance, acid pre-treatment with H_3_PO_4_ or H_2_SO_4_ is especially effective.^43^ Pre-treatments must be carefully calibrated to retain scalability while reducing environmental impact in order to adhere to the principles of green chemistry, and hence, maintaining equilibrium between these two factors is essential.^44^ As one of the most popular engineering thermoplastics in the world, acrylonitrile–butadiene–styrene (ABS) copolymer is prized for its durability, dimensional stability, impact resistance, and processability.^45^ It is extensively utilized in consumer electronics, automobile components, home appliances, and increasingly used 3D printing filaments. As additive manufacturing becomes more widely employed, its popularity is increasing rapidly.^46^ Every year, millions of tons of ABS trash are produced, especially in businesses with short product lifecycles. The conventional mechanical recycling of ABS frequently causes chain scission and property degradation, limiting its potential for high-value reuse.^47–49^ A more viable strategy is chemical treatment: the copolymer structure of ABS, which consists of acrylonitrile, butadiene, and styrene units, offers chemically reactive sites that can be specifically used during pre-treatment and pyrolysis to introduce heteroatom functionalities, adjust electrical conductivity, and create disordered porosity. ABS also has a high intrinsic carbon content.^50,51^

**Fig. 1:**
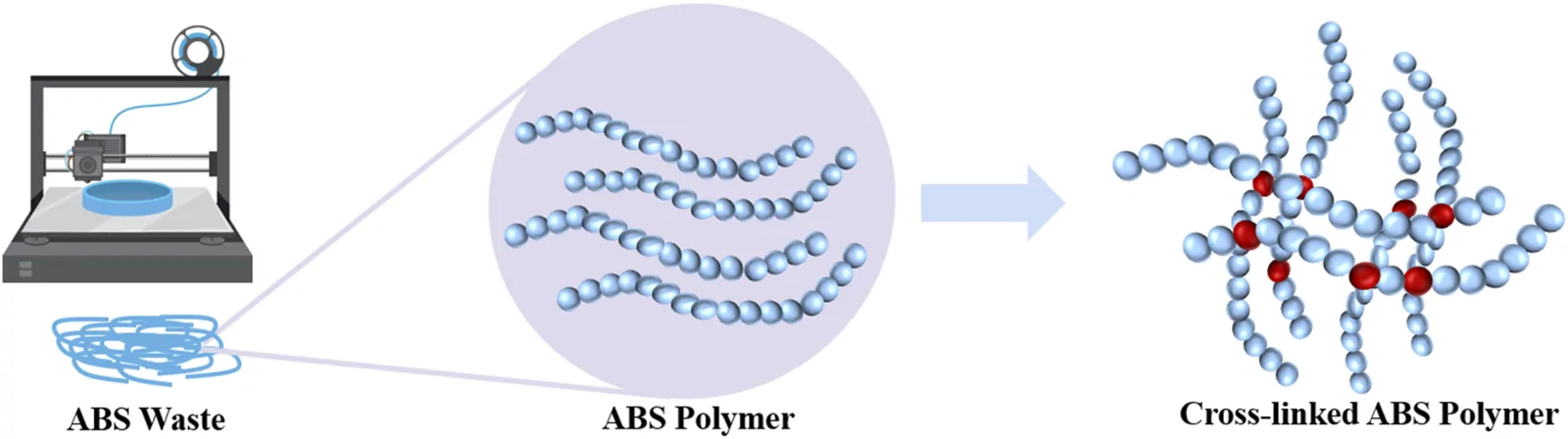
Schematic of the crosslinking phenomenon of acrylonitrile–butadiene–styrene (ABS).

In order to turn ABS trash into carbon anodes for LIBs, this work looks at two corresponding methods: direct carbonization of raw ABS and an acid-assisted pathway that involves H_2_SO_4_ cross-linking before pyrolysis (Fig. 1). Sulfuric acid treatment stabilizes the polymer structure, suggesting that cross-linking transforms the linear ABS structure into a high-molecular-weight structure by connecting linear polymer chains through sulfur groups. This leads to lower loss of the plastic, increased product yield, and improved electrochemical characteristics of the carbon material. Thus, we propose an effective method for converting ABS plastic waste into an anode material for LIBs, providing a route to valorize a waste stream that would otherwise be discarded or subjected to lower-value mechanical recycling.

## Experimental part

### Materials

In this study, commercial ABS plastic waste from local 3D printing operations was used as the carbon precursor. Filament offcuts, support structures removed after printing, and failed prints were used as raw materials. Concentrated sulfuric acid (H_2_SO_4_, 98%, Sigma-Aldrich) was used as the crosslinking agent for chemical pretreatment. The materials used in the electrode fabrication and battery assembly included copper foil (current collector), Celgard 3501 polypropylene separator, acetylene black (Timcal) as the conductive additive, polyvinylidene fluoride (PVDF, Sigma-Aldrich), and *N*-methyl-2-pyrrolidone (NMP, Sigma-Aldrich, slurry solvent). A commercial 1 M lithium hexafluorophosphate (LiPF_6_) solution provided by Sigma-Aldrich was used as the electrolyte, with a solvent composed of ethylene carbonate (EC), dimethyl carbonate (DMC) and diethyl carbonate (DEC) in a volumetric ratio of 1 : 1 : 1.

### Cross linking

**Fig. 2:**
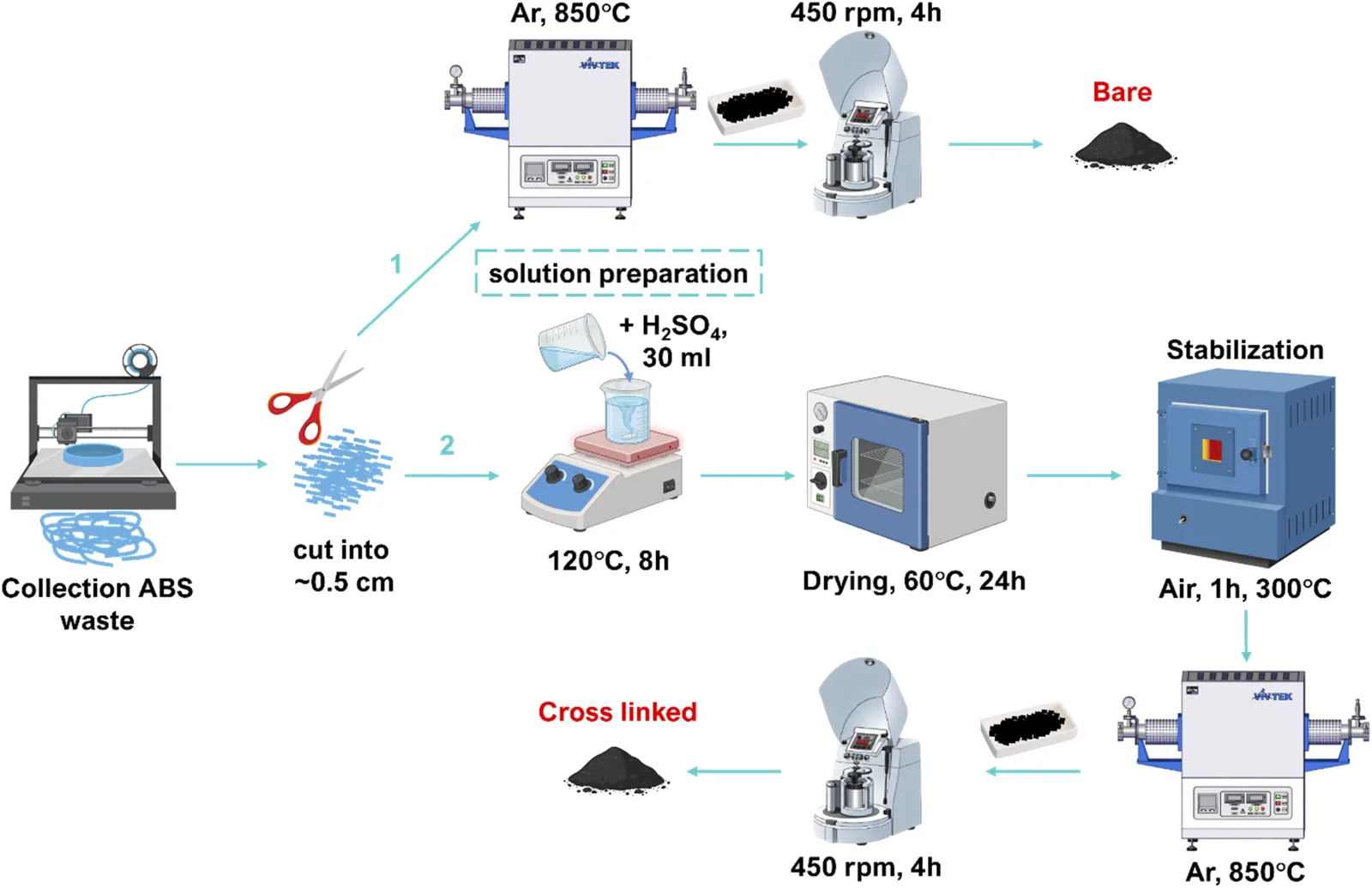
Schematic of the recycling process of acrylonitrile–butadiene–styrene (ABS) plastic waste to convert it into nanostructured carbon anode materials for lithium-ion batteries.

ABS plastic waste was treated with sulfuric acid to introduce sulfonic groups (–SO_3_H) into its structure. These sulfonic acid groups facilitated the crosslinking of the linear polymer chains, which improved the thermal stability of the material and provided additional reactive sites. The electrophilic aromatic sulfonation reaction occurred on the styrene units present in ABS plastic. ABS plastic waste was cut into small pieces of approximately 0.5 mm to facilitate effective acid penetration. The plastic was then washed several times with water and ethanol and dried in an oven at 60 °C for 12 h. This sample was labeled as bare ABS. Next, 20 g of the cut plastic was placed in a glass flask and treated with 30 ml of 98% sulfuric acid. The mixture was stirred at 120 °C for 8 h using a magnetic stirrer. During the crosslinking process, the plastic changed its color from white to black along with an increase in volume, which served as the first visual evidence of crosslinking. After the reaction, the residual acid was decanted, and the sample was washed multiple times with deionized waster until a neutral pH was reached. The obtained crosslinked sample was then dried in an oven at 60 °C for 12 h. After treatment, the sample was labeled as cross-linked ABS. The schematic of the synthesis steps is shown in Fig. 2.

### Synthesis

To obtain carbon materials, both samples were thermally treated. Bare ABS was carbonized in a tube furnace at 850 °C for 2 h under an argon (Ar) atmosphere at a heating rate of 5 °C min^−1^. The dried cross-linked ABS was subjected to an additional stabilization step in a muffle furnace at 300 °C for 1 h in air. This step was introduced to further stabilize the sample and ensure controlled oxidation prior to carbonization. After naturally cooling to room temperature, the cross-linked ABS sample was subsequently carbonized in a tube furnace at 850 °C for 2 h under an argon (Ar) atmosphere at a heating rate of 5 °C min^−1^. After the completion of the heat treatment, both samples were milled using a planetary ball mill for 4 h at 450 rpm. This step was carried out to obtain a homogeneous carbon powder suitable for electrode fabrication.

### Material characterization

Scanning electron microscopy (SEM, JSM IT800, JEOL) and transmission electron microscopy (TEM, JEM-1400Plus, JEOL) were employed to observe the surface morphology and microstructural features. The phase composition and structural ordering were assessed using an X-ray diffractometer (XRD, Rigaku MiniFlex) with an Ni-filtered Cu Kα radiation (*λ* = 1.5406 Å). The interlayer distance (*d*_002_), crystallite height (*L*_c_), and average number of graphene layers (*N*) were calculated from the diffraction peaks of the (002) plane using the Bragg's law and Scherrer equation. A Raman and AFM System (LabRAM, Horiba) was used to investigate the equilibrium between graphitized and disordered carbon domains, and structural defects were quantified from the D-band (∼1334 cm^−1^) and G-band (∼1573 cm^−1^) intensities. The in-plane grain size (*L*_a_) was estimated using the Tuinstra–Koenig relation. The surface chemistry and bonding structure were characterized using X-ray photoelectron spectroscopy (XPS, NEXSA, Thermo Scientific) with a monochromatic Al Kα source (*hν* = 1486.6 eV). Survey and high-resolution spectra were collected in the C 1s, O 1s, N 1s, and S 2p regions. Quantitative elemental analysis (C, H, N, and S) was performed using a CHNS-O elemental analyser (Unicube, Elementar) *via* the combustion method. Energy-dispersive X-ray spectroscopy (EDS) combined with scanning electron microscopy (SEM) yielded spatially resolved elemental distribution maps, confirming the heteroatom distribution in both samples. Using the Brunauer–Emmett–Teller (BET) technique for surface area and the *t*-Plot method for the micro/external surface area and pore volume, the specific surface area and pore texture were ascertained by N_2_ physisorption at 77 K (Micromeritics TriStar II Plus 3.03).

### Electrochemical analysis

The electrochemical performance was evaluated using a CR2032 half-cell. The working electrode was prepared as follows: the obtained carbon material, acetylene black conductive carbon, and PVDF binder were mixed in a weight ratio of 80 : 10 : 10 and dispersed in NMP to form a homogeneous suspension, which was then coated onto a copper foil using a doctor blade. The coated electrode was vacuum-dried at 120 °C for 12 h and then punched into circular discs. The half-cell was assembled in an argon-filled glovebox (MBraun, O_2_ and H_2_O content <0.1 ppm). Metallic lithium chips (Sigma-Aldrich, 99%) simultaneously served as the counter electrode and reference electrode, and a Celgard 3501 polypropylene separator and 1 M LiPF_6_ were used as the separator and electrolyte, respectively. Constant current charge–discharge cycle tests were performed at various current densities using a Neware battery test system. Cyclic voltammetry (CV) was performed using a BioLogic analyzer over a potential range of 0.01–3.0 V (*vs.* Li^+^/Li) at a scan rate of 0.1 mV s^−1^. Electrochemical impedance spectroscopy (EIS) was performed using the same instrument with a sinusoidal perturbation of 5 mV amplitude over the frequency range of 100 kHz to 0.01 Hz. The coated electrode mass, Cu foil mass, and disk area (14 mm diameter, 1.54 cm^2^) were used to calculate the active-material mass loading, which was roughly 1.66 mg cm^−2^ for the bare ABS electrode and 1.56 mg cm^−2^ for the cross-linked ABS electrode. These values correspond to areal capacities of roughly 0.20 and 0.52 mAh cm^−2^, respectively. Although the dried electrode thickness was not directly measured in this study, it is anticipated to fall within the range commonly reported for lab-scale, lightly loaded (1–5 mg cm^−2^) hard-carbon composite electrodes prepared by doctor-blade coating (typically ∼10–30 µm), consistent with values reported for comparable hard-carbon anodes, based on the mass loading achieved here.

## Results and discussion

### Yield and structural characterization

**Fig. 3:**
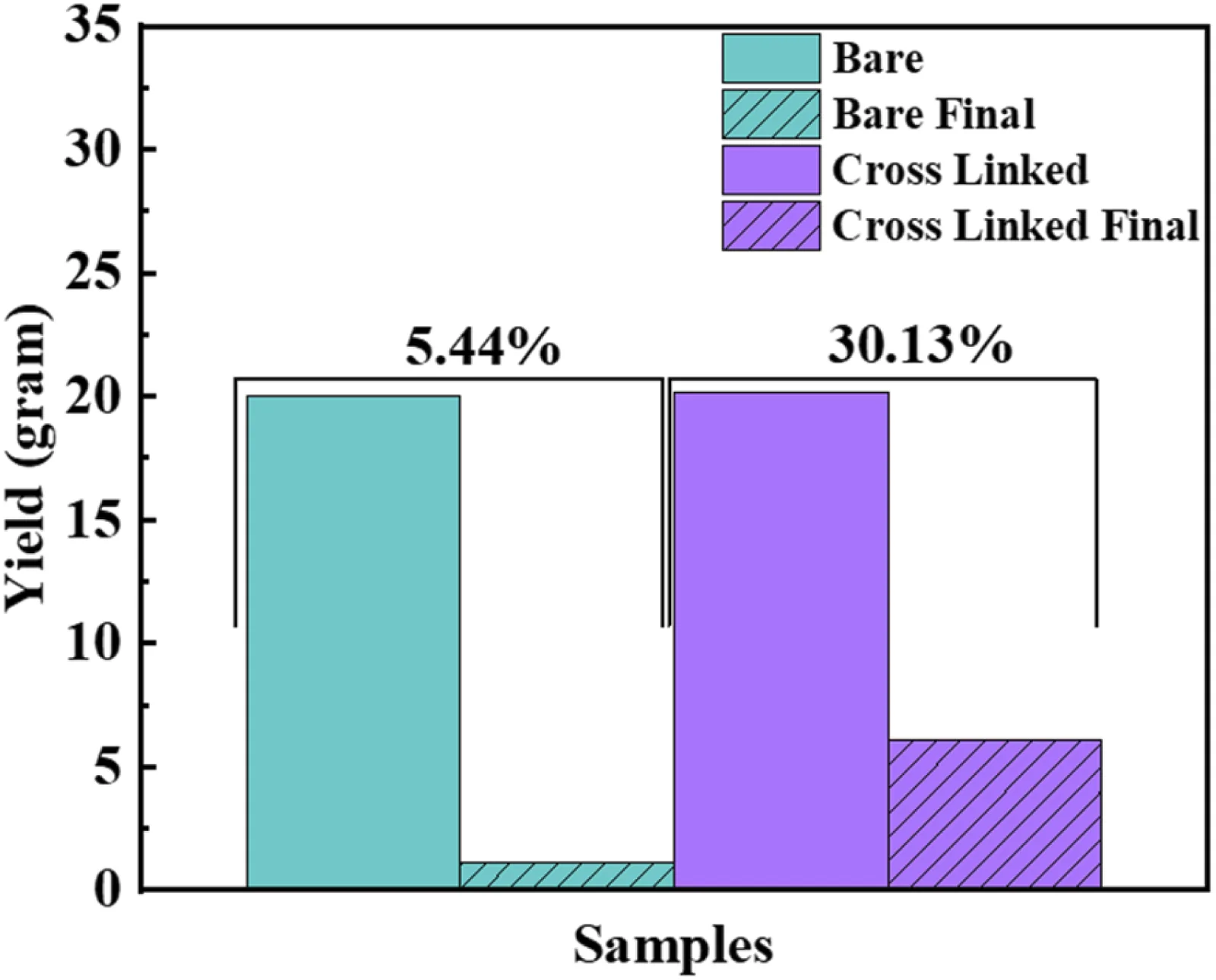
Comparison of the carbon yield before and after pyrolysis for bare ABS and cross-linked ABS. The initial masses of both samples was similar (∼20 g).

The most immediate effect of crosslinking with H_2_SO_4_ is a significant increase in the carbon yield. Both samples had similar initial masses (approximately 20 g), but their behaviour during pyrolysis was radically different. ABS decomposed almost completely, retaining only about 1 g of solid carbon in a yield of just 5.44%. In contrast, the cross-linked sample produced approximately 6 g of solid carbon in a yield of 30.13%. This is almost six times greater than that achieved by the bare ABS (Fig. 3). This substantial increase is attributed to the H_2_SO_4_-induced crosslinking of the polymer prior to thermal treatment. Sulfonation introduces –SO_3_H groups, which link adjacent polymer chains to form a crosslinked network, thus preventing the breakage and loss of volatiles, characteristic of the pyrolysis of bare ABS. Unlike traditional depolymerization and evaporation, the stable backbone undergoes controlled carbonization, resulting in a more cohesive carbon matrix with a higher coke yield and an ordered, pre-graphitized structure. The disordered porous structure formed in this process is not merely a structural feature; it directly opens channels for ion transport and increases the density of available active sites, which together lead to superior electrochemical performance. Therefore, the performance results alone are sufficient to illustrate an important point: acid-catalysed crosslinking can simultaneously improve the efficiency and electrochemical performance of materials.

**Fig. 4:**
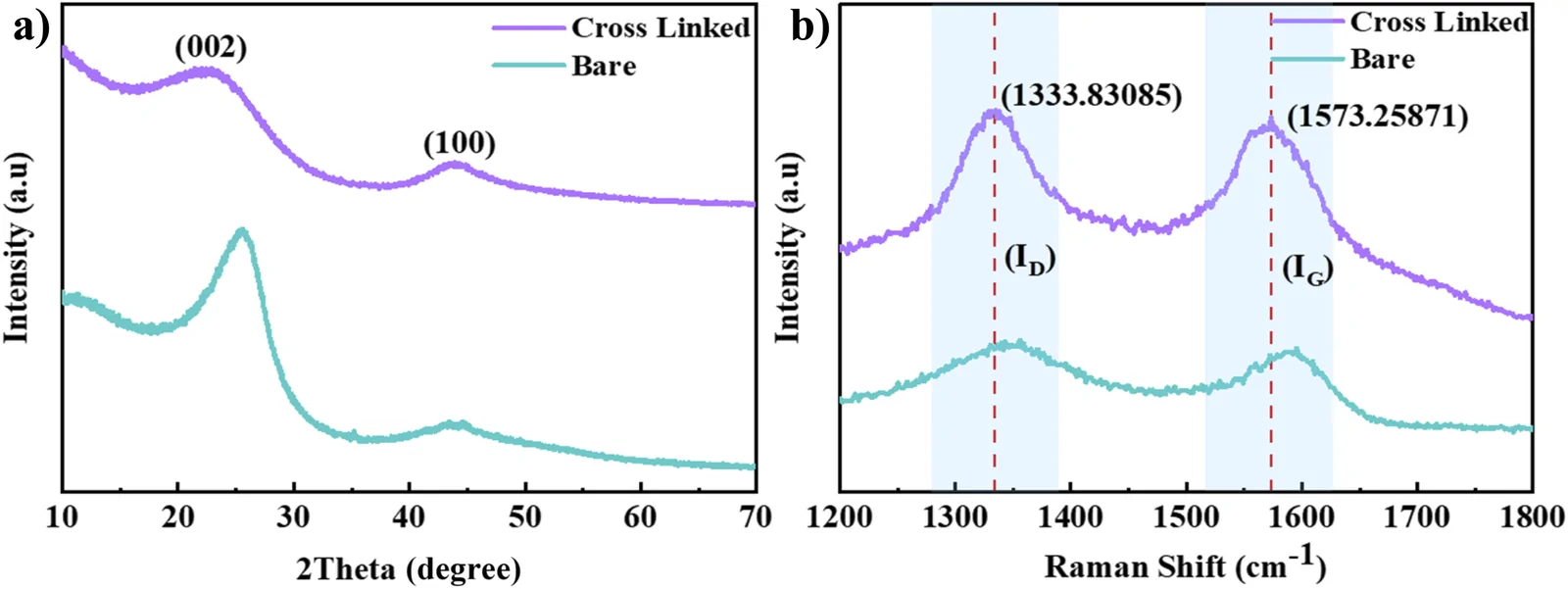
(a) X-ray diffraction (XRD) spectra of the bare ABS- and cross-linked ABS-derived carbon. (b) Raman spectra of the same samples showing the characteristic D and G bands at ∼1334 cm^−1^ and ∼1573 cm^−1^, respectively.

The X-ray diffraction spectra of both samples showed two broad, weak diffraction peaks centred at approximately 2*θ* = 26° and 44° (Fig. 4a), corresponding to reflections from the (002) and (100) crystal planes of the disordered carbon, respectively. The broad peaks and low intensity of these characteristic peaks confirm a turbostratic carbon structure-one that lacks long-range crystalline order but retains short-range stacking similar to graphene. Using Cu Kα radiation (*λ* = 1.5406 Å), the interlayer spacing *d*_002_ was calculated to be 3.7 Å according to Bragg's law (eqn (3.1)). This is significantly larger than the interlayer spacing of 3.35 Å in crystalline graphite. It is well known that expanded interlayer spacing eases lithium-ion insertion by lowering the energy barrier for incorporation.3.1
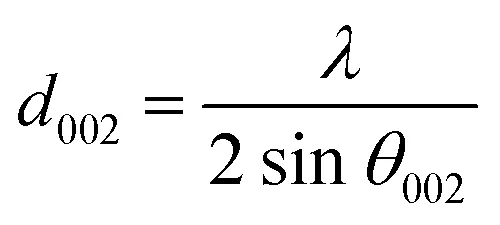


Applying the Scherrer equation (eqn (3.2)) to the (002) reflection, the measured full width at half height (FWHM) (*d*_002_) is 0.86° (0.015 rad), implying that the grain height *L*_c_ is approximately 92.4 Å (9.24 nm). This leads to an estimate of the average number of graphene layers (*N*) of approximately 25 (eqn (3.3)), corresponding to short-range layered domains rather than extended graphitic grains. These parameters, taken together, describe a hard carbon material that is disordered enough to accommodate lithium beyond simple intercalation while possessing enough structure to maintain acceptable conductivity.3.2
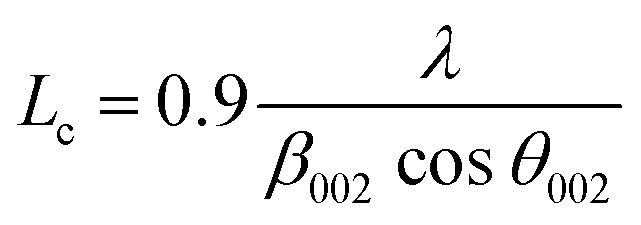
3.3
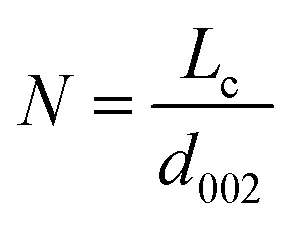


Raman spectroscopy provides a complementary and more sensitive probe of local structural order (Fig. 4b). Both samples exhibit two characteristic bands of disordered graphitic carbon: the D band at ∼1334 cm^−1^, associated with defects and sp^3^-hybridized carbon and the G band at ∼1573 cm^−1^, which arises from the in-plane stretching vibrations of sp^2^-hybridized graphene domains. The crosslinked sample has a slightly higher *I*_D_/*I*_G_ ratio of approximately 1 : 1, indicating a higher density of structural defects than that of the bare ABS-derived carbon. This is attributed to the –SO_3_H groups and heteroatom doping introduced during acid treatment, which disrupt the local graphitic order, although the overall quality of carbonization improves. Far from being a drawback, this defect-rich character is advantageous for lithium storage: the defect sites and edge planes provide additional intercalation sites beyond the ordered graphene layers.

Using the Tuinstra–Koenig relationship (eqn (3.4)), where *λ* = 532 nm and *I*_D_/*I*_G_ ≈ 1.1, the in-plane grain size is estimated to be approximately 17.5 nm, confirming the existence of an ordered graphitic structure, albeit limited to the nanoscale. By combining X-ray diffraction and Raman spectroscopy data, it was concluded that the ABS-derived carbon materials, especially the crosslinked ABS-derived carbon materials, possess large interlayer spacing, nanoscale graphitic domains, and a defect-rich microstructure. It is this combination of structural features that ensures efficient Li^+^ incorporation, stability against volume changes during cycling, and supports the high reversible capacities reported in the Electrochemical Performance Section.3.4
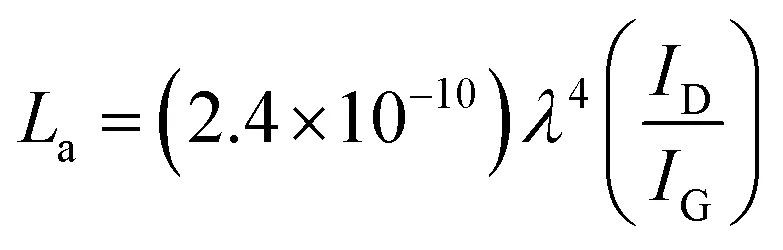


### Morphological characterization

**Fig. 5:**
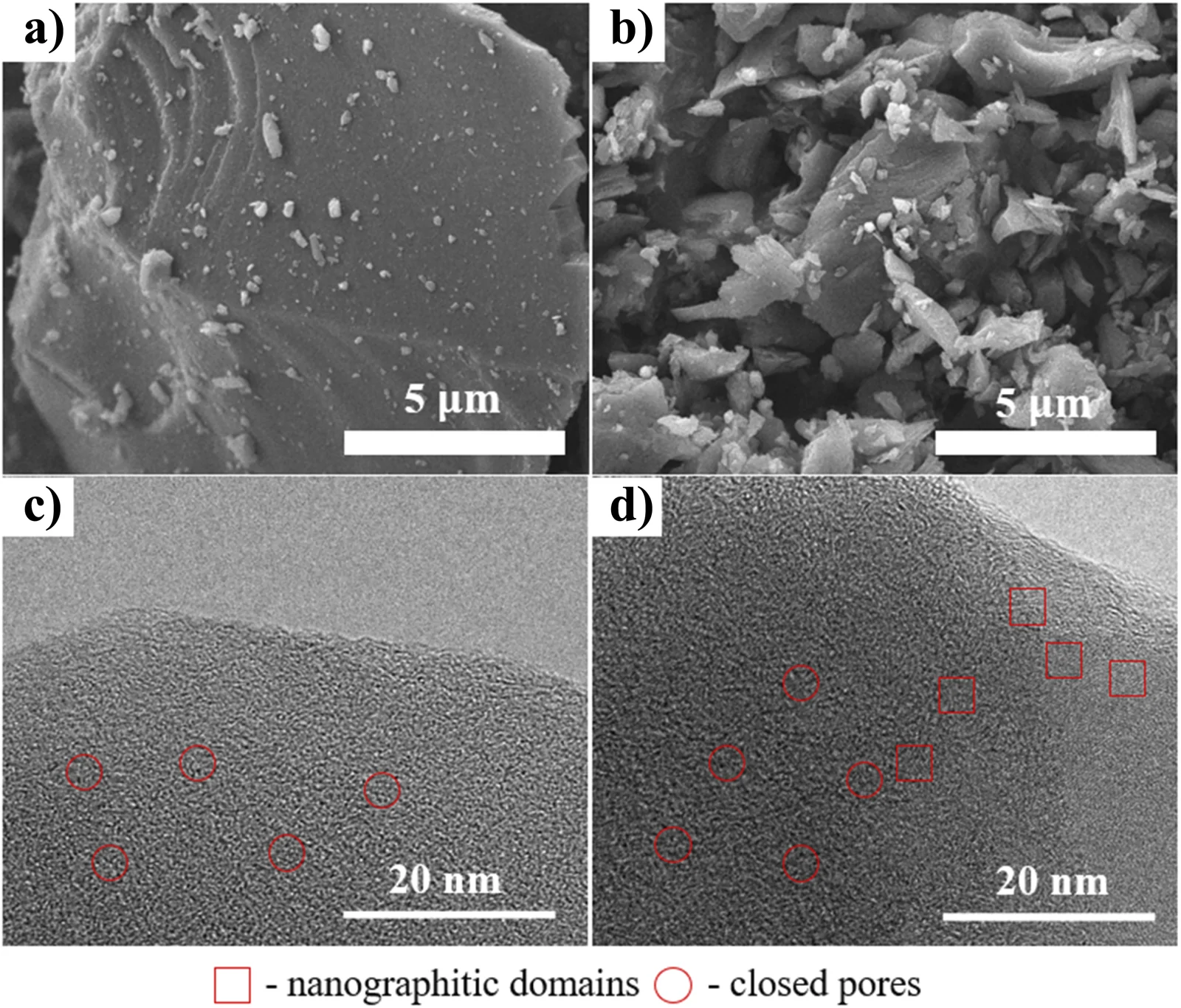
(a) SEM image of the bare ABS-derived carbon, (b) SEM image of the cross-linked ABS-derived carbon, (c) TEM image of the bare ABS-derived carbon, and (d) TEM image of the cross-linked ABS-derived carbon.

The structural differences revealed by X-ray diffraction and Raman spectroscopy become visually apparent when the two carbon materials were observed under an electron microscope. Following the synthesis, the morphological features of the obtained carbon materials were examined to reveal the effect of sulfuric acid crosslinking on their structures. Carbon samples carbonized without crosslinking generally exhibit a relatively smooth, compact, and dense surface with a plate-like or blocky morphology and low porosity (Fig. 5a). These samples tend to have larger particle sizes and are occasionally decorated with a small number of fine particles. In contrast, the cross-linked ABS-derived carbon displays a more fragmented, rough, and coarse morphology with a highly disordered porous structure (Fig. 5b). A noticeable difference in the particle size is also evident between the two samples. These observations suggest that crosslinking significantly affects the morphology of the carbon material. The more disordered porous structure of the cross-linked ABS-derived carbon can enhance ion accessibility and increase the specific surface area, which may contribute to improved electrochemical performance.

To further investigate the microstructure of the carbon materials, TEM analysis was performed, as shown in Fig. 5c and d. The TEM images revealed a significant difference in the distribution of carbon atoms among the samples. In the case of the bare ABS-derived carbon, the structure is predominantly less amorphous, with limited development of graphitic domains. The structure is dominated by closed pores, and the overall morphology appears less ordered. In contrast, the cross-linked ABS-derived carbon exhibits disordered regions, along with partially aligned layers and locally stacked domains. Notably, a higher density of nanographitic domains is observed. The TEM results indicate that sulfuric acid crosslinking leads to a more disordered, nanographitic-domain-rich carbon structure, consistent with the enhanced electrochemical performance discussed below. Direct evidence for improved charge-transport kinetics comes from electrochemical impedance spectroscopy (Fig. 10c), where the cross-linked electrode exhibits a lower charge-transfer resistance (*R*_2_ = 13.86 Ω) than the bare electrode (*R*_2_ = 15.46 Ω), indicating more favorable interfacial kinetics for the cross-linked carbon; however, TEM morphology alone does not directly establish bulk electronic conductivity.

The results of the structural (XRD, Raman) and morphological (SEM and TEM) analyses show that samples crosslinked using H_2_SO_4_ result in robust nanostructured carbons with a microporous structure and amorphous nature, which are highly favourable for better electrochemical performance and increased post carbonization yield.

### Porosity analysis

**Fig. 6:**
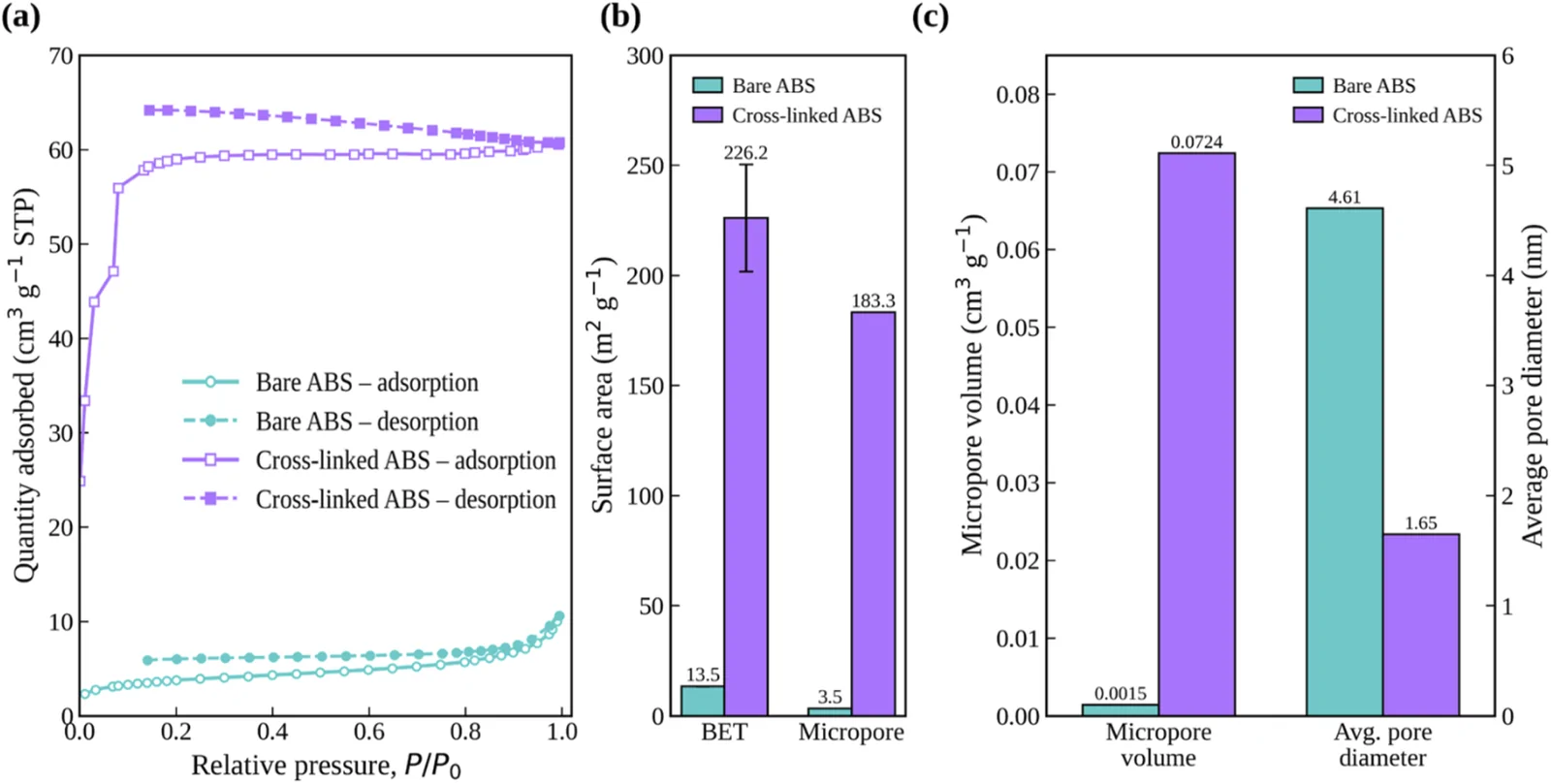
(a) N_2_ adsorption–desorption isotherms, (b) BET/micropore surface area and (c) micropore volume/average pore diameter for bare ABS- and cross-linked ABS-derived carbons. Cross-linking shifts the pore texture from mesopore- to micropore-dominated, increasing the BET surface area to >16-fold (13.5 → 226.2 m^2^ g^−1^) and micropore volume to ∼50-fold (0.0015 → 0.0724 cm^3^ g^−1^).

As illustrated in Fig. 6a–c, both materials underwent N_2_ adsorption–desorption analysis in order to quantitatively confirm the porosity changes indicated by SEM and TEM. The BET specific surface area of the bare ABS-derived carbon is only 13.5 ± 0.04 m^2^ g^−1^. It is mostly composed of mesopores (external surface area = 10.0 m^2^ g^−1^ and average pore width = 4.6 nm using the 4*V*/*A* technique) with a very little micropore contribution (*t*-Plot micropore volume = 0.0015 cm^3^ g^−1^). Prior to carbonization, sulfonation cross-linking causes the pore shape to change from mesopore-dominated to micropore-dominated, increasing the BET surface area to 226.2 ± 24.4 m^2^ g^−1^, a >16-fold increase. The average pore diameter decreases from 4.6 to 1.7 nm, while the *t*-Plot micropore area increases to 183.3 m^2^ g^−1^ (81% of the total BET area) and micropore volume increases to 0.0724 cm^3^ g^−1^, a ∼50-fold increase. This demonstrates that –SO_3_H-mediated cross-linking creates a hierarchically micro-/meso-porous carbon network instead of just roughening the particle surface and is compatible with the fragmented, disordered shape observed in the SEM/TEM images (Fig. 5b and d). Because a larger accessible surface area increases the number of Li^+^ adsorption/intercalation sites and shortens the effective diffusion length into the carbon matrix, the resulting high density of micropores and short diffusion pathways are consistent with the improved initial coulombic efficiency and rate capability and lower charge-transfer/SEI resistance (*R*_2_ = 13.86 Ω and *R*_3_ = 44.84 Ω for the cross-linked ABS-derived carbon *vs.* 15.46 Ω and 60.68 Ω for the bare ABS-derived carbon, respectively; Fig. 10c, reported in the Electrochemical Performance section).

### Surface analysis

Along with morphology and crystalline structure, to understand the actual behaviour of these carbon materials at the electrode–electrolyte interface, it is necessary to know their surface composition: the elements present, their distribution, and the chemical bonds formed. We combined elemental mapping using energy-dispersive X-ray spectroscopy (EDS), X-ray photoelectron spectroscopy (XPS), and elemental analysis by CHNS combustion to evaluate these parameters, and the results were consistent and mutually corroborating. CHNS analysis was employed to determine the elemental composition of the bare ABS- and cross-linked ABS-derived carbon samples. Bare specimens showed only carbon (∼84%) with trace hydrogen (∼0.14%), and no detectable nitrogen or sulfur. In contrast, the cross-linked sample contained ∼3.9% nitrogen and ∼4.0% sulfur, with slightly higher carbon (∼86.7%) and hydrogen (∼1.1%) contents. These results confirm the incorporation of heteroatoms during cross-linking, consistent with the intended chemical modification.

**Fig. 7:**
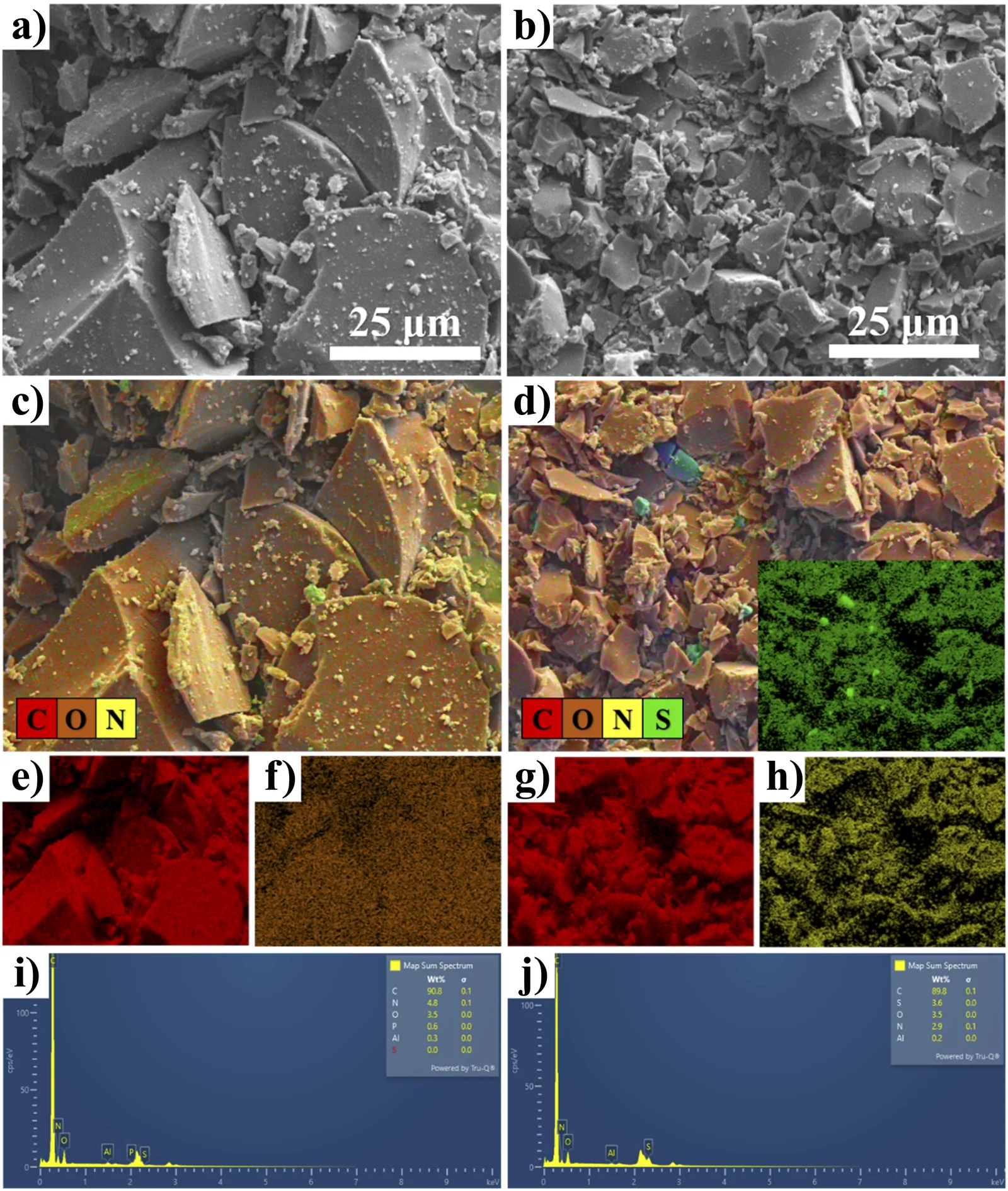
SEM-EDS analysis of carbon materials obtained from ABS plastic. (a) SEM image of the bare ABS-derived carbon; (b) SEM image of the cross-linked ABS-derived carbon; (c) EDS image of the bare ABS-derived carbon; elemental distribution for C, O, and N; (d) EDS image of the cross-linked ABS-derived carbon; elemental distribution for C, O, N, and S; (e) C distribution in the bare ABS-derived carbon; (f) O distribution in the bare ABS-derived carbon; (g) C distribution in the cross-linked ABS-derived carbon; (h) O distribution in the cross-linked ABS-derived carbon; (i) EDS spectra of the bare ABS-derived carbon; and (j) EDS spectra of the cross-linked ABS-derived carbon.

EDS spectroscopy (Fig. 7a–j) revealed direct and significant differences between the two samples. The chemical composition of the bare ABS-derived carbon is relatively simple. EDS spectroscopy (Fig. 7i) confirmed a carbon content of approximately 90.4 wt%, with trace amounts of oxygen (8.5 wt%) and nitrogen (0.6 wt%), likely due to atmospheric exposure and polymer particle residues rather than artificial functionalization. Trace amounts of aluminium and phosphorus were also detected, which were attributed to contamination during the manufacturing process. Sulfur was completely absent. The crosslinked carbon presents a marked contrast. Overlays of the elemental diagrams (Fig. 7d) and individual elemental maps (Fig. 7d, g and h) show that carbon, oxygen, nitrogen, and sulfur are present on the surface of the crosslinked carbon and exhibit a spatial distribution. The presence of sulfur (Fig. 7d) is of particular importance as it provides direct spatial evidence that the –SO3H groups introduced during treatment with H_2_SO_4_ have survived pyrolysis and are actually integrated into the carbon structure, rather than simply being adsorbed onto the surface.

**Fig. 8:**
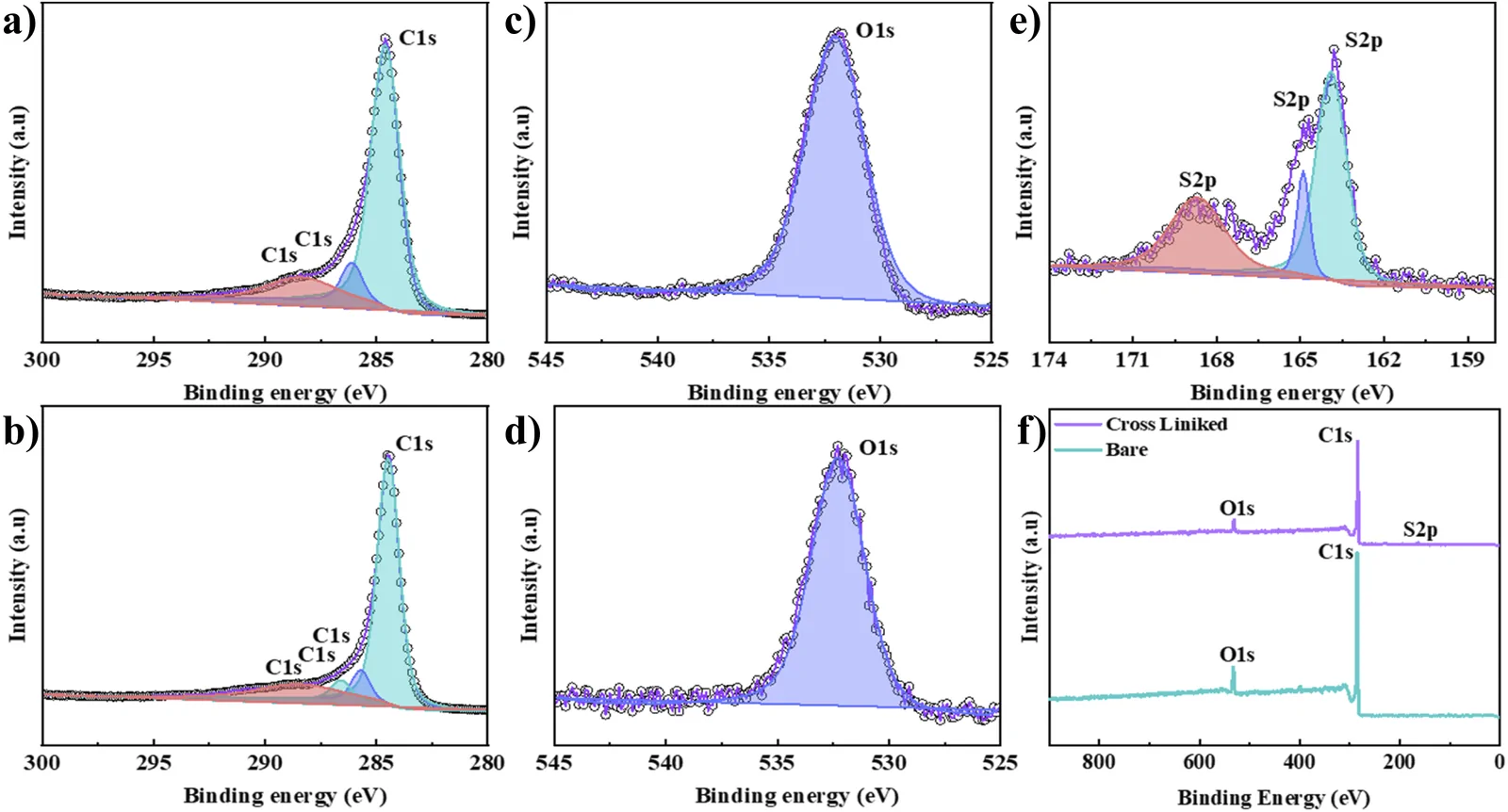
X-ray photoelectron spectroscopy (XPS) results of the bare ABS- and cross-linked ABS-derived carbons. (a and b) High-resolution C 1s spectrum and (c and d) O 1s spectrum of the bare ABS- and cross-linked ABS-derived carbons, (e) S 2p spectrum of the cross-linked ABS-derived carbon, and (f) comparison of the complete XPS spectra of the bare ABS- and cross-linked ABS-derived carbon surfaces.

To analyse the surface chemistry, X-ray photoelectron spectroscopy (XPS) was conducted. As shown in Fig. 8a, the peak at around 285 eV demonstrates the presence of C–C groups, which is significant among the anode material's other peaks; for the cross-linked ABS-derived carbon (Fig. 8b), the peak at around 286 eV shows the presence of hydroxyl groups (C–O/C–O–C), which enhance the pseudocapacitive reactions and improve ion transport. Peaks at around 288–289 eV confirms the availability of carbonyl (C=O) and carboxyl (O–C=O) groups, which are crucial for providing surface active sites, improving kinetic performance and stabilizing the structure to boost the capacity. In Fig. 8c and d, the broad oxygen peaks exhibit the presence of carbonyl (O=C) and carboxyl (O=C–O) groups at around 530–532 eV, while the peaks in the range of 533–533.5 eV demonstrate the presence of hydroxyl groups (O–C), which also help in providing active sites for ion adsorption and facilitating redox activity. Fig. 8e shows high-resolution S 2p XPS spectrum of the cross-linked ABS-derived carbon. The peaks ranging from 163 to 164.5 eV confirm the covalent bonding of sulfur within the carbon matrix, usually attributed as C–S–C structures. These groups not only increase the number of active sites for ion adsorption but also increase the electrical conductivity due to strong π conjugation. Also, C–S–C groups reduce the ion diffusion barrier by enlarging the interlayer spacing (*d*_002_) of the carbonaceous material. Peaks at around 164.5–165.5 eV show the reduced elemental sulfur, which enhances structural stability when incorporated into the carbon matrix. The peak at around 167–169.5 eV shows the C–SO_X_–C groups, which play a critical role in improving the specific capacity by increasing the interlayer spacing similarly to other functional groups. Improving the adsorption of alkali metal ions on the surface, also within the carbon structure, by increasing the surface defects enhances electron transfer and the wettability of the carbon anode surface by the electrolyte, promoting charge transfer and forming a stable SEI layer. The XPS survey spectrum in Fig. 8f demonstrates all the elements present in the respective nanostructure carbon samples. C 1s, O 1s, and S 2p spectra with no detectable impurity peaks imply the robustness of the synthesised materials. Results of the structural analysis techniques (EDS, CHNS, XPS) confirm that the carbon material synthesised using the cross-linked ABS plastic possesses a prominent surface chemistry favourable for its use as an anode material for advanced LIBs.

### Electrochemical performance

**Fig. 9:**
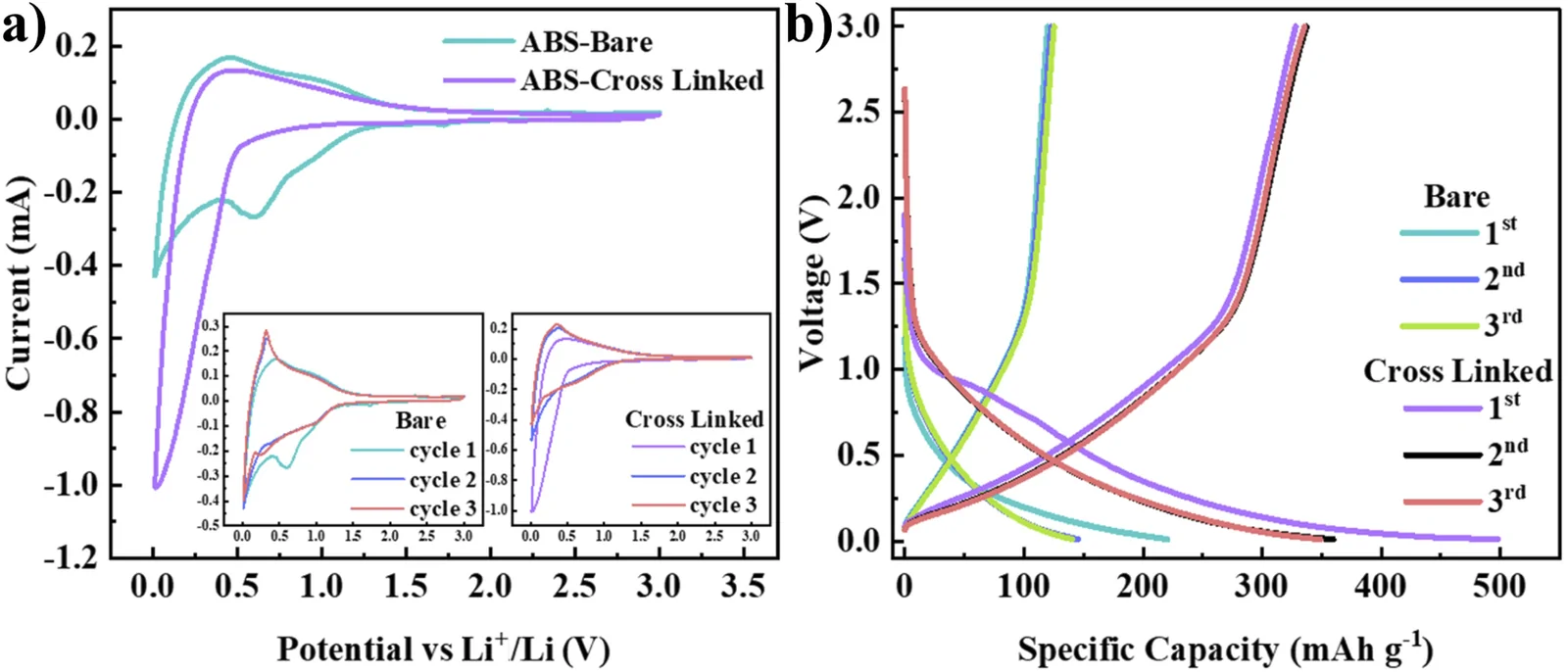
(a) Cyclic voltammetry curves of the anodes based on the bare ABS-derived carbon and cross-linked ABS-derived carbon. (b) Galvanostatic charge and discharge profiles of the anodes based on the bare ABS-derived carbon and cross-linked ABS-derived carbon.

**Fig. 10:**
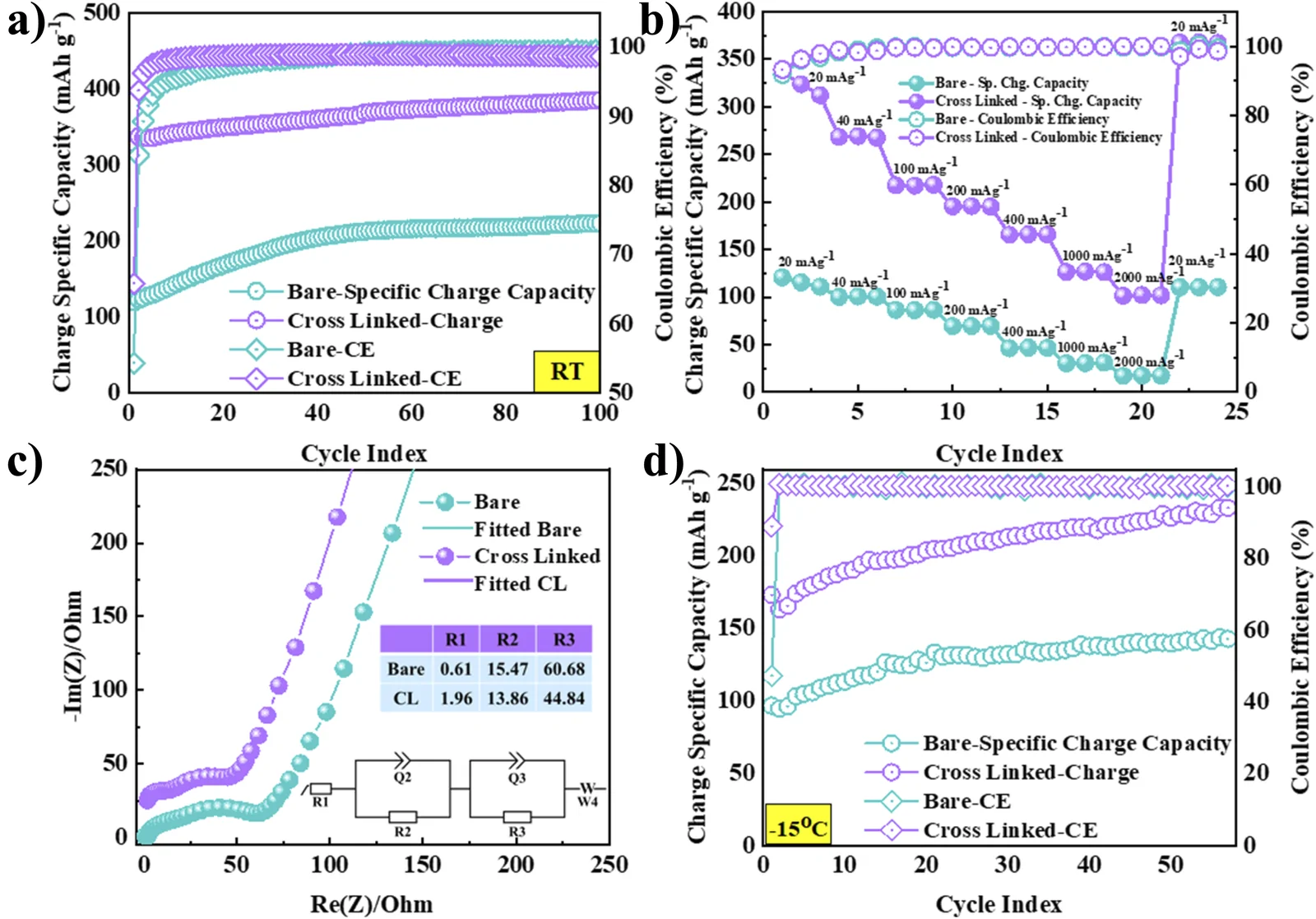
(a) Long-term cycling performance and coulombic efficiency of the bare ABS- and cross-linked ABS-derived electrode after 100 cycles. (b) Rate performance and coulombic efficiency of the bare ABS- and cross-linked ABS-derived electrodes at different current densities (20–2000 mA g^−1^). (c) Electrochemical impedance spectroscopy (EIS) results for the bare ABS- and cross-linked ABS-derived electrodes. (d) Cyclic performance and coulombic efficiency of the bare ABS- and cross-linked ABS-derived electrodes over 57 cycles at −15 °C.

Having established the structural and chemical superiority of the crosslinked ABS-derived carbon, we evaluated its electrochemical characteristics. We performed four types of measurements in a lithium-ion battery half-cell configuration: cyclic voltammetry, electrochemical impedance spectroscopy, galvanostatic charge–discharge curves, and charge/discharge rate tests. The results were fully consistent with the entire predicted characterization data. After structural, morphological and surface characterization, the electrochemical analysis was conducted in half-cell setup. The pronounced imbalance between the cathodic and anodic current responses visible in the cross-linked ABS-derived electrode's first CV cycle (Fig. 9a, inset) reflects irreversible processes concentrated in the initial cycle—principally due to SEI formation and initial electrolyte decomposition—rather than sustained irreversibility; by the 2nd and 3rd cycles (shown in the same inset), the cathodic/anodic response is markedly more balanced, consistent with a stabilizing SEI. The 98% coulombic efficiency reported for the cross-linked ABS-derived electrode (Fig. 10a) is the steady-state value averaged over cycles 2–100 of the galvanostatic test once this initial irreversibility has subsided and should not be read against the first-cycle CV response, which inherently reflects the electrode's least-reversible cycle.

In Fig. 9b, the galvanostatic charge and discharge profiles are presented. The bare ABS-derived anode shows a reduced reversible capacity of ∼100 mAh g^−1^ and high voltage hysteresis, which increases with the number of cycles. Decrease in the discharge capacity is due to the possible slower ion transport and unstable SEI formation. In comparison with the bare ABS-derived anode, the cross-linked ABS-derived anode delivers a reversible capacity of more than 300 mAh g^−1^ with less polarisation and stable cycling. This enhancement in specific capacity is the result of lithiation/delithiation pathways. Crosslinking and the presence of carboxyl, carbonyl and hydroxyl groups increase the wettability and conductivity, along with structural integrity upon repeated cycling. The cyclic voltammetry curves confirm that the cross-linked ABS-derived anode performs better than the bare ABS-derived anode. Similarly, the galvanostatic charge and discharge profiles confirm that the cross-linked ABS-derived anode performs well. According to the galvanostatic profiles in Fig. 9b, the bare ABS-derived electrode's first-cycle discharge and charge capacities were roughly 222 and 120 mAh g^−1^, respectively. This corresponds to an initial coulombic efficiency (ICE) of approximately 54% and a first-cycle irreversible capacity of approximately 102 mAh g^−1^. With an ICE of about 67% and an irreversible capacity of about 165 mAh g^−1^, the cross-linked electrode produced a first-cycle discharge capacity of about 500 mAh g^−1^ and a charge capacity of about 335 mAh g^−1^. In accordance with the more balanced cathodic/anodic CV response observed for the cross-linked ABS-derived sample in Fig. 9a, the higher ICE of the cross-linked ABS-derived electrode, despite its larger absolute irreversible capacity, indicates that its higher initial capacity is recoverable to a great extent upon delithiation.

In Fig. 10a, cyclability of the bare ABS- and cross-linked ABS-derived electrodes at 20 mA g^−1^ is shown. From the initial cycles, the cross-linked ABS-derived electrode outperformed the bare ABS-derived electrode. The cross-linked ABS-derived electrode has a specific charge capacity of more than 300 mAh g^−1^, which is superior to the ∼150 mAh g^−1^ of the bare ABS-derived electrode. Also, the coulombic efficiency of the cross-linked ABS-derived electrode was maintained at ∼98% while that of the bare ABS-derived one reached only ∼95% because of the rapid capacity fading due to poor structural integrity and insufficient Li^+^ accessibility. From these findings, it is clear that the sulfonation of nanostructured carbon results in the rigidness of the carbon matrix and facilitates enough ion transport pathways to maintain the specific capacity during long-term cycling, providing better life cycle for advanced next-generation LIBs.

Rather than being a measuring artifact, the slow rise in reversible capacity observed for both samples over the first cycles in Fig. 10a is consistent with an activation-type mechanism frequently described for disordered, defect-rich hard carbons. Due to incomplete wetting and the still-forming, structurally unstable SEI layer, which prevents Li^+^ from reaching a portion of the available intercalation sites and defect/edge planes, electrolyte access to the interior of the porous, defect-rich carbon network (Fig. 5b and d) is restricted during the first few cycles. Our EIS results (Fig. 10c) clearly show that the cross-linked ABS-derived electrode has a lower charge-transfer resistance (*R*_2_ = 13.86 Ω) and SEI resistance (*R*_3_ = 44.84 Ω) than that of the bare ABS-derived electrode (*R*_2_ = 15.46 Ω, *R*_3_ = 60.68 Ω). This is because the SEI reorganizes into a thinner and more conductive layer as cycling progresses, gradually lowering the interfacial resistance to Li^+^ transport. The additional defect sites and disordered porosity (Fig. 5d) only become electrochemically accessible after this initial break-in period, which is consistent with the lower, more stable *R*_2_/*R*_3_ values, indicating that Li^+^ can access the electrode surface and diffuse into the carbon matrix more readily once the SEI is stabilized. For turbostratic and defect-rich hard-carbon anodes and supports, this activation behavior—in which capacity increases before plateauing rather than instantly achieving its maximum value—has been extensively documented. It does not, however, contradict the structural insights given by XRD, Raman, and TEM.

In Fig. 10b, the rate capability of both the bare and cross-linked ABS-derived electrodes is shown at an extended charge density ranging from 20 mA g^−1^ to 2000 mA g^−1^. When the bare ABS-derived electrode was charged at 20 mA g^−1^, it exhibited a charge capacity of ∼150 mA g^−1^, but an increase in the C-rate resulted in rapid capacity fading at 2000 mA g^−1^. The bare ABS-derived electrode's specific charge capacity dropped to 18 mAh g^−1^ due to the collapsed structural matrix, hindering the ion transport. In parallel, the cross-linked ABS-derived electrode exhibited a robust specific charge capacity of ∼340 mAh g^−1^ at 20 mA g^−1^ and maintained the trend of stability when charged at higher C-rates of up to 2000 mA g^−1^ with a considerable specific charge capacity of ∼140 mAh g^−1^ due to its excellent structural integrity and wide ion transport pathways formed by the sulfonation of the nanostructure carbon.

To investigate the superior electrochemical performance of the bare ABS- and cross-linked ABS-derived electrodes, electrochemical impedance spectroscopy (EIS) was performed. The Nyquist plots of both the ABS-derived samples are presented in Fig. 10c. The difference between the origin and the first semicircle in the high-frequency region represents the internal resistance of the bulk material in the half-cell (*R*_1_). The complete and incomplete semicircles observed in the mid-frequency region correspond to the charge transfer resistance (*R*_2_) and SEI layer resistance (*R*_3_), respectively. In the low-frequency region, the straight line following the semicircles corresponds to the Warburg impedance, which reflects the Li^+^ diffusion capability toward the electrode surface during redox reactions. The equivalent circuit model used to obtain the resistance values (*R*_1_, *R*_2_, and *R*_3_) is presented in Fig. 10c (inset). The cross-linked ABS-derived electrode exhibits lower *R*_2_ (13.86) and *R*_3_ (44.84) values, whereas the bare ABS-derived electrode shows higher *R*_2_ (15.46) and *R*_3_ (60.68) values. A lower *R*_2_ generally indicates more favorable redox reaction kinetics, which is associated with a higher availability of active sites and an enlarged electroactive surface area for Li^+^ intercalation. This resistance is minimized in the cross-linked ABS-derived electrode, which correlates with the predominance of nanopores in its structure. The formation of the SEI layer can be mitigated, and Li^+^ can more easily pass through this layer in systems with a lower *R*_3_ value. The cross-linked ABS-derived electrode demonstrates the lowest *R*_2_ and *R*_3_ values and thus exhibits superior electrochemical performance compared with the bare ABS-derived electrode. The nanoporous structures are beneficial as they provide more accessible active sites, thereby enhancing the redox reaction kinetics. However, excessively small pores can hinder Li^+^ and electrolyte diffusion, ultimately limiting the overall ion transport and electrochemical performance, as observed in the bare ABS-derived electrode. Bare ABS shows a higher *R*_2_, which restricts its kinetic performance and reduces the overall rate of redox reactions. Therefore, the cross-linked ABS-derived electrode is a more effective material as it provides a better balance between ion diffusion and fast reaction kinetics. This balance enables the cross-linked ABS-derived electrode to achieve a higher specific capacity.

In Fig. 10d, the sub-zero temperature (−15 °C) performance of nanostructured carbon is shown. The bare ABS-derived nanostructured carbon electrode exhibited a specific charge capacity of ∼100 mAh g^−1^, while the cross-linked ABS-derived electrode outperformed the bare ABS-derived one with a specific charge capacity of ∼200 mAh g^−1^ in the sub-zero temperature of −15 °C. Along with the initial performance, the cross-linked ABS-derived electrode maintained its capacity up to 57 cycles with considerable coulombic efficiency in comparison to the bare ABS-derived electrode. This robust capacity retention shows that the disordered porosity and sulfonation by H_2_SO_4_ cross-linking preserve the ion transport pathways and conductivity even at sub-zero temperatures, showing considerable performance where conventional carbons fail.

The advancement shown here is inherent to the chemistry of ABS itself rather than the sulfonation stage alone, even though acid pre-treatment of plastic waste before carbonization is a tried-and-true method. Any heteroatom functionality beyond sulfur must be introduced through an additional, separate doping treatment, such as a subsequent ammonia or urea treatment, to introduce nitrogen since previous plastic-to-carbon routes using PET, PP, or PS rely on backbones that are either purely aliphatic (PP) or present only a single sulfonatable moiety (PS, and the aromatic ring in PET). In contrast, ABS is a terpolymer in which the acrylonitrile block is a precursor that contains nitrogen, and the styrene block offers the same electrophilic aromatic site for H_2_SO_4_ sulfonation used in PS-derived carbons. Therefore, without the need for a separate nitrogen-doping stage, a single acid treatment step applied to ABS produces a carbon that is concurrently co-doped with both sulfur and nitrogen (∼4.0 wt% S and ∼3.9 wt% N by CHNS analysis; Fig. 7 and 8). The unique contribution of this work in comparison to previous PET-, PP-, and PS-to-carbon studies is the one-step N,S co-doping, which arises directly from the terpolymer composition rather than from process complexity, and is, to our knowledge, not achievable from single-heteroatom plastic feedstocks under the same treatment.

## Conclusions

In this century, plastic waste and the demand for battery materials are two of the major challenges faced by materials science. This study demonstrates a route by which ABS waste can be diverted from landfills and valorized into a functional battery component, though realizing this at scale will require a comprehensive evaluation of the process's own resource and energy demands. We converted 3D-printed ABS plastic waste into nanostructured carbon anodes by H_2_SO_4_ crosslinking and pyrolysis, achieving an almost sixfold increase in carbon yield (from 5.44% to 30.13%) while producing a material with the structural and chemical properties required for high-performance lithium-ion batteries. The crosslinking pretreatment resulted in the formation of sulfonic groups and a disordered porous structure, as confirmed by XPS and CHNS analyses. The resulting carbon material exhibits an increased interlayer spacing of 3.7 Å and nanoscale graphitic domains of approximately 17.5 nm, both of which are favourable for efficient Li^+^ intercalation. The electrochemical performance was satisfactory for all tested parameters. The crosslinked ABS-derived anode demonstrated an initial coulombic efficiency of approximately 67% (discharge/charge capacity of ≈500/335 mAh g^−1^) and a specific capacity exceeding 300 mAh g^−1^ in the first cycle, and it retained approximately 350 mAh g^−1^ after 100 cycles with a coulombic efficiency of approximately 98%, along with retaining approximately 140 mAh g^−1^ at a current density of 20 mA g^−1^. It is important to highlight that these benefits did not diminish at low temperatures: at −15 °C, the crosslinked anode retained a capacity close to 200 mAh g^−1^ and a coulombic efficiency close to 100% after 57 cycles, demonstrating stability that is unattainable with untreated carbon materials. In conclusion, this study confirms that acid-assisted treatment is a robust method for converting ABS plastic—one of the fastest-growing plastic waste streams worldwide—into a high-performance anode material; scalability and net environmental benefit will depend on further optimization of chemical consumption, energy input, and acid recovery. Future work will focus on validating the full cell system, BET porosity characterization (to quantify the hierarchical pore structure), and systematically optimizing the crosslinking conditions to further improve the performance and electrochemical characteristics.

## Author contributions

Aisha Zhanaikhan: conceptualization, investigation, methodology, data curation, formal analysis, visualization, writing – original draft. Kuanysh Kurtibay: data curation, formal analysis. Aliya Mukanova: resources, validation, writing – review & editing. Kim Sung-Soo: resources, validation. Arailym Nurpeissova: supervision, project administration, writing – review & editing.

## Conflicts of interest

There are no conflicts to declare.

## Data Availability

All data supporting the findings of this study are available within the paper.
